# 2499. What Are We Waiting for? Chronic Hepatitis C Treatment Initiation During Inpatient Admission

**DOI:** 10.1093/ofid/ofad500.2117

**Published:** 2023-11-27

**Authors:** YoungYoon Ham, HaYoung Ryu, Carissa Williams, Jane Babiarz, Cara D Varley, Amber C Streifel, Marissa Maier, Michael Conte, Sujeet Govindan

**Affiliations:** Oregon Health & Science University, Portland, Oregon; Oregon Health & Science University Hospital and Clinics, Portland, Oregon; Oregon Health & Science University, Portland, Oregon; Oregon Health & Science University, Portland, Oregon; Oregon Health and Science University, Portland, Oregon; Oregon Health and Science University, Portland, Oregon; VA Portland Health Care System/Oregon Health and Sciences University, Portland, OR; Oregon Health and Science University, Portland, Oregon; Oregon Health & Science University, Portland, Oregon

## Abstract

**Background:**

Chronic hepatitis C virus (HCV) infection is highly prevalent among people who inject drugs. This vulnerable population experiences many barriers to engaging with the healthcare system. Evaluating and treating HCV during a hospital stay can be an opportunity to cure and prevent adverse HCV complications in a population that can be difficult to reach in an outpatient setting.

**Methods:**

This was a retrospective review of treatment outcomes of patients who started on treatment for HCV infection with direct acting antivirals (DAAs) while hospitalized at an academic medical center between January 2022 and January 2023. DAAs were dispensed by outpatient pharmacies and brought into the hospital for patient use. Various teams were involved in the treatment of these patients and level of support varied between groups, ranging from full social services and pharmacy support to follow-up clinic visits only. Data collected included demographics, insurance type, injection drug use, housing status, DAA agents used, treatment duration and completion status, and sustained virological response (SVR) 12 or more weeks post-treatment.

**Results:**

A total of 17 patients began treatment with DAAs while hospitalized. Sixteen (94%) were treated with glecaprevir-pibrentasvir for 8 weeks, while 1 (6%) received sofosbuvir-velpatasvir for 12 weeks. Sixteen (94%) reported using injection drugs and 12 (71%) reported unstable housing. All patients had government-managed health insurance, with 16 having Medicaid. Twelve (71%) patients completed treatment: 9 of 12 patients who completed treatment attained SVR12 and 3 were lost to follow up after treatment completion. Of the 5 patients who did not finish treatment, 2 attained SVR12 despite incomplete therapy, 1 is not yet due for SVR12, and 2 were lost to follow up.

**Figure:**
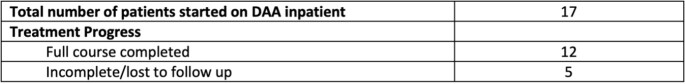

**Conclusion:**

In a vulnerable population with barriers to outpatient care, in-hospital evaluation and DAA initiation is a valuable opportunity to cure. Further evaluation is needed to optimize and implement strategies for inpatient HCV treatment initiation.

**Disclosures:**

**All Authors**: No reported disclosures

